# Supplementary material for: Association of miR-499 Polymorphism and Its Regulatory Networks with Hashimoto Thyroiditis Susceptibility: A Population-Based Case-Control Study
Source: Int J Mol Sci. 2021 Sep 18;22(18):10094. doi: 10.3390/ijms221810094 (PMC8470033; doi:10.3390/ijms221810094)
Supplement: Supplementary file 1 [file ijms-22-10094-s001.zip › Supplementary Table S1-selected genes.pdf]

Supplementary Table S1. Selected nodes based on initial analyses for PPIN, miR-TF and co-expression regulatory networks. Nodes with the highest degree were used for new networks reconstruction using the STRING (v11.5) database.

Label	Degree	Betweenness
**From initial PPIN**		
MEOX2	174	105725.3
SDCBP	83	62693.4
UBE2D3	71	58615.64
MAPK8	71	52068.96
NFKBIA	47	29486.37
HSPA1L	45	33307.74
IVNS1ABP	41	31631.25
MAD2L1	40	43529.36
UBE2E3	34	15498.3
TCF7L2	20	13988.14
PLS3	19	16845.91
**From initial co-expression**		
GPR65	37	666
TAGAP	1	0
MYO1F	1	0
MAP4K1	1	0
CD5	1	0
ARHGAP9	1	0
CD48	1	0
VAV1	1	0
IL10RA	1	0
FYB	1	0
PSTPIP1	1	0
IKZF1	1	0
CD84	1	0
NCKAP1L	1	0
**From initial miR-TF network**		
ZIC2	292	220452.09
NFKBIA	102	71452.21
RAP2C	76	29506.18
HOXA5	72	33168.41
TCF7L2	58	33137.3
EYA1	57	19536.4
UBE2D3	50	28115.07
STIM2	50	19542.13
ZFAND5	48	16422.28
MEOX2	47	20024.36
