# Supplementary material for: Association of miR-499 Polymorphism and Its Regulatory Networks with Hashimoto Thyroiditis Susceptibility: A Population-Based Case-Control Study
Source: Int J Mol Sci. 2021 Sep 18;22(18):10094. doi: 10.3390/ijms221810094 (PMC8470033; doi:10.3390/ijms221810094)
Supplement: Supplementary file 1 [file ijms-22-10094-s001.zip › Supplementary Table S2-PPIN-pathways.pdf]

Supplementary Table S2. Summary of functional enrichment in the network generated from initial PPIN selected genes (for complete details, see Supplementary File 4).

Description	Observed	Background	Strength	FDR
**Biological process (GO)**				
Proteasome-mediated ubiquitin-dependent protein catabolic process	10	323	1.29	3.02E-07
Proteolysis	15	1256	0.88	3.42E-07
Macromolecule catabolic process	13	1058	0.89	5.19E-06
Organonitrogen compound catabolic process	13	1070	0.88	5.40E-06
Regulation of canonical Wnt signaling pathway	6	291	1.11	0.0016
I-κB kinase/NF-κB signaling	4	74	1.53	0.0017
NIK/NF-κB signaling	4	81	1.49	0.0022
Interleukin-1-mediated signaling pathway	4	96	1.42	0.0034
β -catenin-TCF complex assembly	3	29	1.81	0.0034
Stress-activated MAPK cascade	4	107	1.37	0.0047
**Molecular function (GO)**				
β-catenin binding	6	86	1.64	3.18E-05
Ubiquitin conjugating enzyme activity	4	40	1.8	0.0017
Transcription factor binding	8	672	0.88	0.0089
Ubiquitin protein ligase binding	6	296	1.11	0.0089
Enzyme binding	13	2239	0.56	0.0122
**Local network cluster (STRING)**				
Canonical NF-κB pathway, and NF-κB/Dorsal	4	12	2.32	0.0449
**KEGG pathway**				
Ubiquitin mediated proteolysis	8	135	1.57	2.05E-08
Cell cycle	7	120	1.57	2.13E-07
Wnt signaling pathway	5	154	1.31	0.00027
MAPK signaling pathway	5	288	1.04	0.0025
Th1 and Th2 cell differentiation	3	87	1.34	0.0073
IL-17 signaling pathway	3	92	1.31	0.0082
NF-κB signaling pathway	3	101	1.27	0.0095
Toll-like receptor signaling pathway	3	101	1.27	0.0095
Th17 cell differentiation	3	101	1.27	0.0095
T cell receptor signaling pathway	3	101	1.27	0.0095
**Reactome pathway**				
Adaptive Immune System	11	743	0.97	0.00012
Signal Transduction	18	2741	0.62	0.00012
Immune System	16	1956	0.71	0.00012
CLEC7A (Dectin-1) signaling	6	96	1.6	0.00012
Antigen processing: Ubiquitination & Proteasome degradation	8	304	1.22	0.00012
Inactivation of APC/C via direct inhibition of the APC/C complex	4	20	2.1	0.00015
FcεRI signaling	5	129	1.39	0.0021
Innate immune system	10	1025	0.79	0.0024

Interleukin-1 family signaling	5	136	1.37	0.0024
Activation of NF-κB in B cells	4	65	1.59	0.0034
FCERI mediated NF-κB activation	4	80	1.5	0.0058
Degradation of beta-catenin by the destruction complex	4	81	1.49	0.0059
TRAF6 mediated induction of NF-κB and MAP kinases upon TLR7/8 or 9 activation	4	89	1.45	0.0071
Toll Like Receptor 3 (TLR3) Cascade	4	92	1.44	0.0072
Interleukin-1 signaling	4	99	1.41	0.0073
**Protein domains and features (InterPro)**				
NF-κB/Dorsal	3	5	2.58	0.0035
Ubiquitin-conjugating enzyme E2	4	38	1.82	0.0035
Ubiquitin-conjugating enzyme, active site	4	25	2	0.0035
Rel homology domain, conserved site	3	5	2.58	0.0035
NF-κB IPT domain	3	5	2.58	0.0035
Rel homology domain (RHD), DNA-binding domain	3	10	2.28	0.005
Rel homology dimerisation domain	3	10	2.28	0.005
Rel homology domain (RHD), DNA-binding domain superfamily	3	10	2.28	0.005
Ubiquitin-conjugating enzyme/RWD-like	4	53	1.68	0.0062
