# Supplementary material for: Association of miR-499 Polymorphism and Its Regulatory Networks with Hashimoto Thyroiditis Susceptibility: A Population-Based Case-Control Study
Source: Int J Mol Sci. 2021 Sep 18;22(18):10094. doi: 10.3390/ijms221810094 (PMC8470033; doi:10.3390/ijms221810094)
Supplement: Supplementary file 1 [file ijms-22-10094-s001.zip › Supplementary Table S3-coexpression-pathways.pdf]

Supplementary Table S3. Summary of functional enrichment in the network generated from initial co-expression network selected genes (for complete details, see Supplementary File 5).

Description	Observed	Background	Strength	FDR
**Biological process (GO)**				
Immune system process	14	2481	0.76	3.12E-05
Immune response	11	1588	0.85	0.00037
Regulation of response to stimulus	15	4114	0.57	0.00067
Immune response-activating cell surface receptor signaling pathway	5	311	1.22	0.0448
Regulation of immune system process	8	1514	0.74	0.0448
Positive regulation of immune system process	7	949	0.88	0.0448
Signal transduction	14	4876	0.47	0.0448
Positive regulation of catalytic activity	8	1489	0.74	0.0448
Positive regulation of GTPase activity	5	407	1.1	0.0448
Leukocyte activation	7	929	0.89	0.0448
Regulation of immune response	7	896	0.91	0.0448
Antigen receptor-mediated signaling pathway	4	199	1.32	0.0448
Positive regulation of T cell activation	4	209	1.29	0.0448
Leukocyte migration	5	316	1.21	0.0448
**Local network cluster (STRING)**				
Mixed, incl. Generation of second messenger molecules, and Antigen activates B Cell Receptor (BCR) leading to generation of second messengers	6	38	2.21	1.17E-05
Mixed, incl. T cell receptor complex, and CD28 dependent Vav1 pathway	4	22	2.27	0.0091
**KEGG pathway**				
Natural killer cell mediated cytotoxicity	4	121	1.53	0.0026
Yersinia infection	4	125	1.52	0.0026
T cell receptor signaling pathway	3	101	1.49	0.0201
**Reactome pathway**				
Immune system	11	1956	0.76	0.0062
Generation of second messenger molecules	3	29	2.03	0.0328
**Protein domains and features (InterPro)**				
SH2 domain	4	112	1.57	0.0468
SH3 domain	5	221	1.37	0.0468
SH3-like domain superfamily	5	211	1.39	0.0468
SH2 domain superfamily	4	113	1.56	0.0468
