# Supplementary material for: Association of miR-499 Polymorphism and Its Regulatory Networks with Hashimoto Thyroiditis Susceptibility: A Population-Based Case-Control Study
Source: Int J Mol Sci. 2021 Sep 18;22(18):10094. doi: 10.3390/ijms221810094 (PMC8470033; doi:10.3390/ijms221810094)
Supplement: Supplementary file 1 [file ijms-22-10094-s001.zip › Supplementary Table S4-miR-TF-pathways.pdf]

Supplementary Table S4. Summary of functional enrichment in the network generated from initial miR-TF selected genes (for complete details, see Supplementary File 6).

Description	Observed	Background	Strength	FDR
**Biological process (GO)**				
Positive regulation of cellular biosynthetic process	16	2005	0.89	4.04E-09
Positive regulation of transcription, DNA-templated	14	1587	0.94	2.39E-08
Interleukin-1-mediated signaling pathway	6	96	1.79	4.58E-07
I-κB kinase/NF-κB signaling	5	74	1.82	8.10E-06
Negative regulation of cell death	10	999	0.99	8.19E-06
NIK/NF-κB signaling	5	81	1.78	1.13E-05
Negative regulation of apoptotic signaling pathway	6	232	1.4	4.45E-05
Regulation of type I interferon production	5	130	1.58	7.45E-05
Fc-epsilon receptor signaling pathway	4	115	1.53	0.0011
Regulation of canonical Wnt signaling pathway	5	291	1.23	0.0015
Response to cytokine	8	1101	0.85	0.0012
Pattern recognition receptor signaling pathway	4	125	1.5	0.0013
Positive regulation of protein ubiquitination	4	126	1.49	0.0014
Regulation of innate immune response	5	301	1.21	0.0017
Negative regulation of NF-κB transcription factor activity	3	85	1.54	0.0077
Toll-like receptor signaling pathway	3	100	1.47	0.0109
**Molecular function (GO)**				
Transcription regulatory region sequence-specific DNA binding	9	1028	0.93	0.0007
RNA polymerase II transcription regulatory region sequence-specific DNA binding	8	878	0.95	0.0007
DNA-binding transcription activator activity, RNA polymerase II-specific	7	449	1.18	0.0007
Chromatin binding	7	570	1.08	0.0007
DNA-binding transcription factor activity	9	1238	0.85	0.0007
Transcription factor binding	8	672	1.07	0.0007
Ubiquitin protein ligase binding	6	296	1.3	0.0007
Transcription regulator activity	11	1657	0.81	0.0007
β-catenin binding	3	86	1.53	0.0189
**Local network cluster (STRING)**				
Canonical NF-kB pathway, and NF-κB/Dorsal	6	12	2.69	4.45E-08
**KEGG pathway**				
Chemokine signaling pathway	5	186	1.42	5.62E-05
RIG-I-like receptor signaling pathway	4	70	1.75	5.62E-05
B cell receptor signaling pathway	4	78	1.7	5.62E-05
Th1 and Th2 cell differentiation	4	87	1.65	6.47E-05
IL-17 signaling pathway	4	92	1.63	6.59E-05
NF-κB signaling pathway	4	101	1.59	7.65E-05
Toll-like receptor signaling pathway	4	101	1.59	7.65E-05
T cell receptor signaling pathway	4	101	1.59	7.65E-05
TNF signaling pathway	4	112	1.54	8.84E-05

**Reactome pathway**				
Activation of NF-κB in B cells	6	65	1.96	1.18E-06
IκBA variant leads to EDA-ID	4	7	2.75	1.79E-06
Interleukin-1 signaling	6	99	1.77	3.25E-06
FcεRI mediated NF-κB activation	5	80	1.79	2.77E-05
TRAF6 mediated NF-κB activation	4	24	2.21	2.77E-05
Toll Like Receptor 3 (TLR3) Cascade	5	92	1.73	3.42E-05
Downstream TCR signaling	5	91	1.73	3.42E-05
TRAF6 mediated induction of NF-κB and MAP kinases upon TLR7/8 or 9 activation	5	89	1.74	3.42E-05
TAK1 activates NF-κB by phosphorylation and activation of IKKs complex	4	32	2.09	3.44E-05
CLEC7A (Dectin-1) signaling	5	96	1.71	3.44E-05
TRIF(TICAM1)-mediated TLR4 signaling	5	96	1.71	3.44E-05
NF-κB is activated and signals survival	3	13	2.35	0.00024
Adaptive Immune System	7	743	0.96	0.0023
Signaling by WNT	5	296	1.22	0.0041
Immune System	9	1956	0.65	0.0173
**Protein domains and features (InterPro)**				
NF-κB/Dorsal	3	5	2.77	0.0015
NF-κB IPT domain	3	5	2.77	0.0015
Rel homology domain (RHD), DNA-binding domain	3	10	2.47	0.0019
Rel homology dimerization domain	3	10	2.47	0.0019
Rel homology domain (RHD), DNA-binding domain superfamily	3	10	2.47	0.0019
p53-like transcription factor, DNA-binding	3	44	1.82	0.0467
